# The role of counseling on modern contraceptive utilization among HIV positive women: the case of Northwest Ethiopia

**DOI:** 10.1186/s12905-018-0603-3

**Published:** 2018-07-04

**Authors:** Bilen Mekonnen Araya, Abayneh Akililu Solomon, Kahsay Zenebe Gebreslasie, Temesgen Worku Gudayu, Kiber Temesgen Anteneh

**Affiliations:** 1 School of midwifery, college of medicine and health sciences, university of Gondar, Gondar, Ethiopia; 2 Department of midwifery, college of health sciences, Mekelle university, Mekelle, Ethiopia

**Keywords:** Modern contraceptive, HIV positive, Ethiopia, Amhara region

## Abstract

**Background:**

Over 90% of the children with Human Immunodeficiency Virus are infected through the mother to child transmission process according to literatures. Preventing unintended pregnancy by using contraceptive methods is crucial for reducing maternal and child mortality and morbidity. Here we set out to assess the prevalence and associated factors of modern contraceptive utilization among sexually active reproductive age women attending Anti-Retroviral Therapy clinics in Amhara Region referral hospitals in 2016.

**Methods:**

An institution based cross sectional study was carried out from April to July 2016 surveying women of reproductive age attending the Anti-Retroviral Therapy clinics in the five Amhara Region referral hospitals. A pretested and semi-structured questionnaire was used to collect data. EpiInfo7 and SPSS version 20 soft wares were used for data entry and analysis, respectively. Significant associations were identified on the basis of the adjusted odds ratio, with 95% Confidence Interval, and *p* value ≤0.05, was taken as statistically significant.

**Result:**

The proportion of women utilizing modern contraceptives was 47.7% with (95% CI: 43, 52.5%), the male condom being the most (64.2%) utilized method. The use of the contraceptive methods was most prevalent among women 15–24 years of age (AOR = 6.5, 95% CI: 2–10) and age of 25–34 (AOR = 3, 95% CI: 1.6–5.5), having an urban residence (AOR = 0.095, 95% CI: 0.03–0.28), having discussed contraceptives with partner (AOR = 7, 95% CI: 5.3–11.9), receiving counseling from health care providers (AOR = 4.8, 95% CI: 1.8–7), previous history of contraceptive utilization (AOR = 5.6, 95% CI: 2.6–8.3), and with CD4 count >500mm^3^/dl (AOR = 2.4, 95% CI: 1.3–4.3).

**Conclusion:**

The proportion of women utilizing contraceptive has been low in Amhara Region referral hospitals. Encouraging patients to discuss about contraception with partners and repeated counseling by health care providers may strengthen contraceptive utilization.

## Background

Modern contraceptive methods such as tubal ligation, implants, oral contraceptives and injectables are technological advances to reduce the risk of pregnancy and to enable partners to have sexual intercourse at any mutually-desired time [1]. Worldwide, around 150,000 children are newly diagnosed with Human Immunodeficiency Virus (HIV). Over 90% of children are infected via mother-to-child transmission (MTCT). If untreated, about half of infected children will die before their second birthday [2]. The target of reducing new pediatric HIV infections by 90% reflects the impact of the four-prong strategy for the prevention of mother to child transmission (PMTCT) of HIV. It represents not only the effects of the reduction of vertical transmission from an infected mother to her child but also the effects of the reduction of HIV incidence in women of reproductive age and the effects of the reduction of unintended pregnancies among women with HIV all contributing to reducing the number of new pediatric HIV infections [3, 4].

The Sustainable Development Goal aims to end Human Immunodeficiency Virus/Acquired Immune Deficiency Syndrome (HIV/AIDS) epidemics and ensure universal access to family planning methods to all [5]. Preventing unintended pregnancy among HIV-positive women constitutes a critical and cost-effective approach to primary PMTCT of HIV and is a global public health priority for addressing the desperate state of maternal and child health in HIV hyper-endemic settings [6]. An estimated 225 million women who want to avoid pregnancy are not using an effective contraceptive method. In developing regions, an estimated 74 million unintended pregnancies occur every year. If all unmet needs for modern methods were met, 52 million of these unintended pregnancies could be averted, thereby preventing the deaths of 70,000 women from pregnancy related causes [7]. HIV-infected pregnant or post-partum women are eight times more mortality than their uninfected counterparts [8].

According to studies conducted in Denmark, India, and Nepal, 75, 95, and 70.8% of the HIV infected women were using contraceptive methods respectively [9–11]. In South Africa, current use of modern contraceptives was 89.8 and 84% among HIV positive women. Besides, injectables were used by 70% and female sterilization by 7.1% of such women. The most common reason for utilization of such alternatives were the convenience of the methods and the recommendation of care providers [12, 13]. On the other hand, research done in Addis Ababa showed that 71% women reported that they were using contraception. Two other studies done in Ethiopia Tigray Region showed that 46.3 and 44.3% of women were using modern contraceptive methods, which was comparable to 47.9% reported in Debremarkos, Ethiopia [14–17].

According to the 2016 Ethiopian demographic and health survey (EDHS) 35% of currently married and 55% of the sexually active unmarried women use modern contraceptives. Amhara Region had 46.9 and 17.4% of modern contraceptive prevalence and unmet need for contraceptives among currently married women respectively [18]. Even though contraceptive methods are supplied for free in Ethiopia, HIV positive women still have unintended pregnancies.

Given the complications and large magnitude of unwanted pregnancy and MTCT of HIV, this research tries to assess the contraceptive prevalence and factors associated with among HIV positive reproductive age women in Amhara region. This research will help to update existing knowledge, inform policy makers and program designers to develop and disseminate tailored reproductive health services to HIV positive women in the study area.

## Methods

### Design, setting and population

This Institution based cross-sectional study was conducted from April to July 2016 at Amhara Region referral hospitals Anti-Retroviral Therapy (ART) clinics. The region has five referral hospitals, namely Debrebirhan, Debremarkos, Felegehiwot, Desse and Gondar. The region’s population was projected to be over 20 million by 2015, which was about 25% of the total population of the Federal State of Ethiopia, and 50.2% were female. According to EDHS 2011, the Amhara Region had 1.6% HIV prevalence, 2.2% among females. An estimated 380,924 people live with the virus in the region, and a total of 12,450 reproductive age women were registered in the ART clinics of the five referral hospitals mentioned above.

### Eligibility criteria

Sexually active HIV positive reproductive age (15–49) women attending ART clinic who had at least one follow up history and willing to allow medical record review for the purposes of confirming the CD4 level were included.

Pregnant and known infertile women or those who had hysterectomy were excluded.

### Sampling and measurement

A sample size of 426 was determined using single population proportion formula by taking account of proportion (P) of 47.9% [16], 95% confidence interval (CI), 5% margin of error (d) and 10% non-response rate. A systematic random sampling technique was used to select the study participants. The total number of reproductive age women were estimated by taking the client flow in the past 3 months to the ART clinics. The sampling interval (K) was determined by dividing the total number of women attending in the ART clinic (N) by the sample size determined for each hospital (n).

The dependent variable was current utilization of modern contraceptive methods and the independent variables were Socio-demographic variables including, age, marital status, place of residence, educational status of women and occupation of women. Service related variables include counseling on contraceptive methods, availability and accessibility of methods. Client related factors including number of living children, future fertility desire, knowledge of contraceptive methods, discussion with partner, previous contraceptive utilization history and HIV related variables which include CD4 level, ART/ pre ART, disclosure of HIV status to partner.

### Operational definitions

Utilization of modern contraceptive methods is using either oral contraceptives, injectables, implants, Intra Uterine Contraceptive Device (IUCD), tubal ligation, vasectomy or male condoms methods for the purpose of delaying, spacing, or limiting pregnancy at the time of data collection.

Knowledgeable about contraceptives: those who mentioned at least one modern contraceptive method [19].

Sexually Active: A client who had sexual intercourse in the last 30 days [20].

### Data collection and analysis

Data were collected via face to face interview using a semi-structured questionnaire, and chart review to assess CD4 level. Five diploma and five BSc graduated midwives working in departments other than the ART clinics were selected to collect data and supervise at each of the five hospitals, respectively. The questionnaire was adapted from different literatures and contextualized, and was also validated by experts on the area. It includes all the factors mentioned in the variable list like socio-demographic and client related factors. The questionnaire was prepared in English and translated to Amharic (local language) and back into English for consistency purpose. Data collectors and supervisors received 1 day of training on how to collect and handle the data. Before implementing the survey, the questionnaire was pre-tested on 22 individuals (5% of the sample) in Debretabor hospital to check the accuracy of the response and to estimate the time needed for interview. Based on the pretest, appropriate modifications on question arrangement and wording were made before the actual data collection. Each day, the collected data were checked for completeness and consistency by the supervisors. The principal investigator monitored the performance of data collectors and supervisors, closely. Participants were taken to an empty room away from the ART clinic to protect privacy and no personal identification information was taken. The collected data were reviewed and checked for completeness before data entry. The returned copies of the questioner were checked for completeness manually.

Then the questionnaires were entered in to Epi info version 7, coded, cleaned and exported to SPSS version 20 for analysis. Frequency tables and graphs were used to summarize the descriptive statistics. Bivariate analysis was used primarily to check which variable had association with the dependent variable individually. Statistical association was checked by 95% CI and crude odd ratio. The variables (P- value of ≤0.2) observed in the bivariate analysis were subsequently included in multivariable analysis. Finally the variables which had significant associations were identified on the basis of the adjusted odds ratio, with 95% CI, and *p* value ≤0.05, was taken as statically significant Table 4.

### Ethical consideration

Ethical clearance was obtained from the Institution at Review Board of the University of Gondar, College of Medicine and Health Sciences. The study commenced after a permission letter was obtained from the five referral hospitals. Informed verbal consent was secured from the study participants. Each respondent was informed about the objective of the study and assurance of confidentiality as the information they gave was not communicated for other people. Privacy was maintained for all participants. We informed the participants of the study that they could quit their participation at any stage without any restrictions.

## Result

### Socio-demographic characteristics

A total of 426 participants took part in this study with a response rate of 98.8%. Out of the participants, 50.8% were 25–34 years old (mean age 32.6 (± 6.4 SD)). The majority, 90.5% live in urban areas, and 99.5% were ethnically Amhara. A large number of the respondents, 79.1% were Orthodox Christians, and 50.6% were married. Over a quarter, 28.2% had primary school education, and 42.5% were housewives (Table 1).

**Table 1:** Socio-demographic characteristics of sexually active HIV positive reproductive age women attending ART clinics in Amhara region referral hospitals, Northwest Ethiopia, 2016 (*n* = 421)

Variables	Frequency	Percent %
Age		
15–24	33	7.8
25–34	214	50.8
≥ 35	174	41.4
Residence		
Urban	381	90.5
Rural	40	9.5
Ethnicity		
Amhara	419	99.5
Tigre	2	0.5
Religion		
Orthodox	333	79
Muslim	84	20
Protestant	4	1
Marital status		
Married	213	50.6
Single	50	11.9
Divorced	81	19.2
Widowed	77	18.3
Educational status		
No formal education	151	35.9
Primary education	119	28.2
Secondary and above	151	35.9
Occupation		
Student	15	3.6
Farmer	12	2.8
Government employed	53	12.6
Daily laborer	53	12.6
Merchant	76	18.1
House wife	179	42.5
Private employed	33	7.8

### HIV and service related factors

Out of the 421 participants, more than half 55.5% have been living with HIV for more than 60 months. Almost all were taking ART drugs, 99.3, and 91% disclosed their status to their partners and 5.7% did not do so, because they were afraid their partners would leave/ divorce them. Almost half 49.45% of the participant partners were HIV positive. Approximately 86.9% received counseling from health care providers about contraceptive methods (Table 2).

**Table 2:** HIV and Fertility related profile of sexually active HIV positive reproductive age women attending ART clinics in Amhara region referral hospitals, northwest Ethiopia 2016 (*n* = 421)

Variables	Frequency (N)	Percent (%)
Time since HIV diagnosis (months)		
< = 60 months	188	44.7
> 60 months	233	55.3
On ART		
Yes	418	99.3
No	3	0.7
Recent CD4 count(mm)		
≤ 500	234	55.6
> 500	187	44.4
HIV status disclosed to partner (*n* = 266)		
Yes	242	91
No	24	9
Partner HIV status (*n* = 266)		
Positive	208	78.2
Negative	29	10.9
I don’t know	29	10.9
Total number of living children		
None	77	18.3
1 or 2	246	58.4
3 or 4	78	18.5
> =5	20	4.8
Last pregnancy intended (*n* = 344)		
Yes	283	82.3
No	61	17.7
Fertility desire (*n* = 421)		
Yes	168	39.9
No	228	54.2
I don’t know	25	5.9

*CD4* Cluster differentiation 4, *ART* Anti-retroviral therapy

### Client related factors

**Fig. 1:**
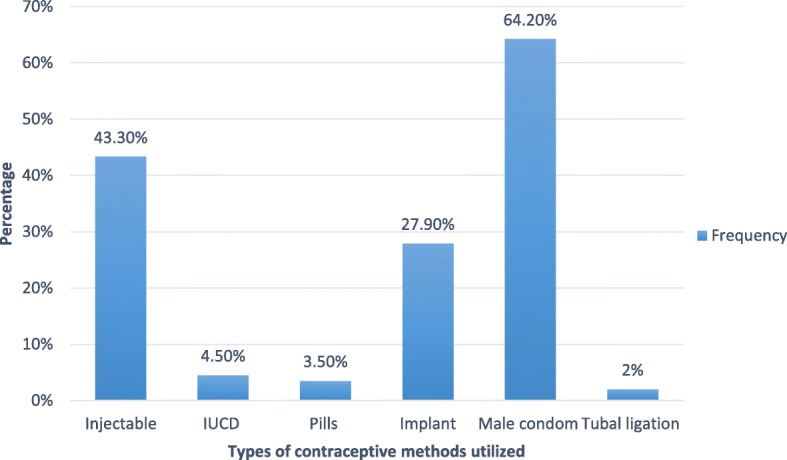
Types of modern contraceptives utilized by sexually active HIV positive reproductive age women attending ART clinics in Amhara region referral hospitals, Northwest Ethiopia 2016

Out of all participants, 81.7% had one or more live children, and out of those who had children 82.3% stated that their last pregnancies were intended. Out of all participants, 54.2% had no future fertility desire; 26.8, and 12.4% of women made the decision because they were poor and they were afraid the children might be infected with HIV, respectively. Out of those who wanted to have children in the future, 33% said they wished sustain to sustain their heredity. Out of all participants, 98.1% were knowledgeable about modern contraceptive methods, and 75.8% of participants had used modern contraceptive methods before, with 67.9% of women used injectables. Almost half 47.7%, were utilizing modern contraceptive methods at the moment, with male condoms 64.2% and injectables 43.3% being the most frequent method used Fig. 1.

**Fig. 2:**
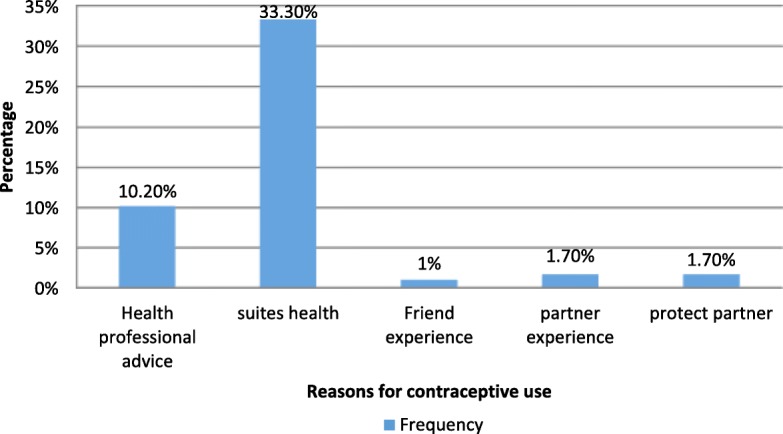
Reasons for modern contraceptive utilization among sexually active HIV positive reproductive age women attending ART clinics in Amhara region referral hospitals, Northwest Ethiopia 2016

**Fig. 3:**
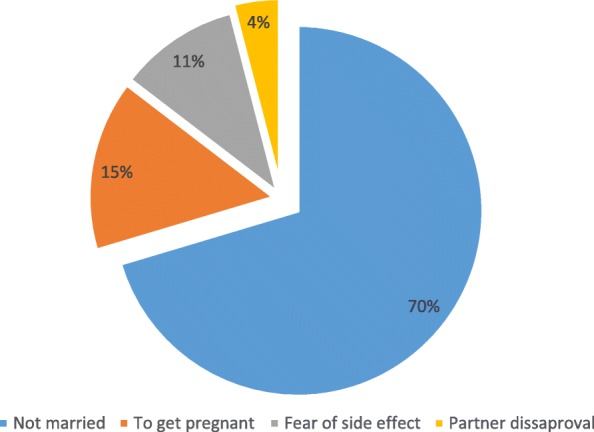
Reasons for not utilizing modern contraceptive methods among sexually active HIV positive reproductive age women attending ART clinics in Amhara region referral hospitals, northwest Ethiopia 2016

Out of the users, 33.3% chose the method because it suited their health, and 37.1% were provided contraceptives from Family Guidance Association/family planning clinics in the hospitals Fig. 2. The main reason for not utilizing modern contraceptives was not being married followed with wanting to get pregnant (Fig. 3).

Out of those whom are utilizing contraceptives, almost all 47.5 and 47.3% responded methods were accessible and available whenever they wanted respectively. Among those who were not currently utilizing contraceptive methods, 34.7% had no intention to use one in the future (Table 3).

**Table 3:** contraceptive utilization and knowledge related profiles of sexually active HIV positive reproductive age women attending ART clinics in Amhara region referral hospitals, northwest Ethiopia 2016 (*n* = 421)

Variables	Frequency (n)	Percent (%)
Knowledge on contraceptive		
knowledgeable	413	98.1
Not Knowledgeable	8	1.9
counseling about methods		
Yes	366	86.9
No	55	13.1
Previous history of utilization		
Yes	319	75.8
No	102	24.2
Previous Type of method utilized (*n* = 319)		
Injectable	216	67.9
IUCD	13	4.1
Pills	94	29.6
Implants	69	21.7
Condom	44	13.8
Current contraceptive utilization		
Yes	201	47.7
No	220	52.3
Place of method received (*n* = 201)		
The ART clinic	35	8.3
FGA/FP clinic in the hospital	156	37.1
Private clinic and pharmacies	9	2.1
Reason to use contraceptives (*n* = 201)		
To limit number of children	95	22.6
To space	106	25.2
Intention to use in the future (*n* = 221)		
Yes	57	13.5
No	146	34.7
I don’t know	17	4
Discussion with partner (*n* = 266)		
Yes	221	52.5
No	45	10.7

### Factors associated with modern contraceptive utilization

In the bivariate logistic regression, age, residence, marital status, recent CD4 level, future fertility desire, counseling on modern contraceptive methods, previous contraceptive utilization history, discussion with partner about HIV and contraceptive methods were associated with modern contraceptive utilization. In multivariable logistic regression analysis, age, residence, marital status, recent CD4 level, counseling on modern contraceptive methods, previous contraceptive utilization history and discussion with partner about HIV and contraceptive methods were associated with modern contraceptive utilization.

The study showed that women aged 15–24 and 24–34, years were 6.5 and 3 times more likely to use modern contraceptive methods compared to those who were ≥ 35 (AOR = 6.5, 95% CI; 2, 21) and 25–34 (AOR = 3, 95% CI; 1.6, 5.5), respectively. In addition, women who had received counseling from health care providers and discussed with their partners were 4.8 and 13 times more likely to use contraceptive methods than those who had no counseling and discussions with their partner (AOR = 4.8, 95% CI; 1.8, 13.03) and (AOR = 13, 95% CI; 5.3, 31.9), respectively. Similarly, those who had history of previous contraceptive utilization were more likely to use modern contraceptive methods than those who had no history (AOR = 5.6, 95% CI; 2.6, 12.3), and women who had recent CD4 count of >500mm^3^/dl were 2.4 times more likely to use than those had CD4 level ≤ 500 (AOR = 2.4, 95% CI; 1.3, 4.3). However, urban dwellers were 90% less likely to use the service than rural dwellers (AOR = 0.095, 95% CI; 0.03, 0.28). Future fertility desire was a confounder for modern contraceptive utilization (Table 4).

**Table 4:** Bivariate and multivariable analysis of factors associated with modern contraceptive utilization among sexually active reproductive age women attending ART clinic in Amhara region referral hospital, Northwest Ethiopia, 2016 (*n* = 421)

Variables	Modern contraceptive utilization		Odds ratio (OR) (95% CI)		
	Yes	No	Crude OR (95% CI)	Adjusted OR (95% CI)	*p*-value
Age					
15–24	20	13	3.4 (1.5, 7.3)	6.5 (2, 21)	0.002
25–34	127	87	3.2 (2.1, 4.9)	3 (1.6, 5.5)	0.000
≥ 35	54	120	1	1	
Residence					
Urban	173	208	0.36 (0.18, 0.72)	0.095 (0.03, 0.28)	0.000
Rural	28	12	1	1	
Marital status					
Married	155	58	1	1	
Single	18	32	0.2 (0.1, 0.4)	1.7 (0.5, 5.5)	0.35
Divorced/Widowed	28	130	0.08 (0.04, 0.13)	0.4 (0.2, 1.06)	0.065
Fertility desire					
Yes	94	74	1.7 (1.17, 2.57)	0.5 (0.3, 1)	0.059
No/I don’t know	107	146	1	1	
Discussion with partner					
Yes	168	53	16 (9.8, 26)	13 (5.3, 31.9)	0.000
No/No partner	33	167	1	1	
Received counseling					
Yes	191	175	4.9 (2.4,10)	4.8 (1.8, 13.03)	0.002
No	10	45	1	1	
Previous contraceptive utilization					
Yes	184	135	6.8 (3.87,12)	5.6 (2.6, 12.3)	0.000
No	17	85	1	1	
CD4 count					
≤ 500	98	136	1	1	
> 500	103	84	1.7 (1.1, 2.5)	2.4 (1.3, 4.3)	0.002

## Discussion

The use of family planning methods is very important for PLWHIV due to the risk of unwanted pregnancy, super infection with new strains of HIV and transmission of HIV from mother to child. The issue of contraceptive use among women enrolled in ART clinics has an important implication for the health of the women and their newborns. In this study, 47.7% of the women were utilizing modern contraceptive methods. This finding is in line with those studies conducted in different regions of Ethiopia reported 44 to 51% [14–16, 21] . Our findings also corresponds to the results of investigations carried out in Rwanda (43%), Kenya (43%) and Ghana (42.6%) [22–24]. That is, perhaps because of the similarities of the characteristics of the populations and research designs.

However, the utilization of contraceptives among postpartum HIV-positive women reported as in Uganda (68%), Zambia (59.5%), and Kenya (61%), were more than ours (47.7%). This might be due to the fact that the study in Zambia included a prospective cohort that assessed utilization after counseling was provided, which might have increased utilization. The studies in Uganda and Kenya included other contraceptive methods including, withdrawal and lactational amenorrhea and were conducted in postpartum period which might reflect higher contraceptive use [25–27]. A study done in South Africa (89.8%) had a higher contraceptive utilization compared to our findings. This is perhaps a different source population and included women who had received antenatal care during their most recent pregnancies which could have increased the percentage because the women could get repeated counselling on postpartum contraceptive methods; they also used convenient sampling methods that could help the researchers to find women who utilized the service better [12].

Other studies including Denmark (75%), Botswana (66%), Nepal (70.8%), India (95%), and Uganda (87.3%) reported much higher utilization than ours. These findings could be explained in relation to a variety of factors. Denmark which had a different population characteristics used a prospective cohort design like Botswana. Nepal employed the snowball sampling technique, while Uganda and India assessed both men and women as a source population, included other contraceptive methods and compared contraceptive utilization before and after HIV diagnosis in which case repeated counseling and awareness creation might have affected practice [9–11, 28, 29].

Other studies in Malawi (17.7%), Uganda (25%), Swaziland (17.3%) and Uganda (34%) reported less than ours. The difference with Uganda, for instance could be the difficulty of accessing facilities and the unavailability of methods in the clinics owing to prolonged conflicts in the country. The difference in time of data collection and high fertility desire among the population could be another reason [30–33].

The study showed that women who were in the 15–24 and 25–34 years of age groups were 6.5 and 3 times more likely to use modern contraceptive methods compared to older ones, respectively. This finding is line with those of done in Zambia and Ethiopian Region [14, 27]. This might be due to in this study, around 60.6% of the women aged 15–24 were secondary school students. Also more than 60% were knowledgeable about modern contraceptive methods. To the contrary studies in Malawi and Ethiopian region showed that older women utilized contraceptive methods more than younger ones. This difference might be due to different socio-cultural factors and sample sizes [32, 34]. Urban dwellers were 90% less likely to use modern contraceptive methods compared to rurals. This finding was similar with EDHS report which showed that 60% of rural women used contraceptive methods, while only 50% of urban women utilized modern contraceptive methods. This shows that, due to previous history of less use in rural areas, the government of Ethiopia stepped up its efforts in terms of awareness creation and improvement of the availability and accessibility of methods [18].

Also, women who had open discussions with their sexual partners about HIV/AIDS and contraceptive methods were 13 times more likely to use modern contraceptive methods than those who had no discussions. The finding is in line with those of studies done in Kenya and different regions of Ethiopia [15, 17, 35, 36]. This shows that open discussions with partners encourage the disclosure of HIV status, planning future fertility desires, and generally speaking it helps to manage life very easily. Similarly, a study in Ugandan HIV clinics indicated that women who did not disclose their HIV status to sexual partners and did not discuss fertility issues were less likely to use contraception [25]. On the other hand, women who had counseling from health care providers about modern contraceptive methods were 4.8 times more likely to use contraceptive methods than those who had no counseling. This finding is supported by those of studies done in India, Nepal, South Africa and Ethiopia [10–12, 37]. Counseling empowers and enables participants to make informed decisions. Such informed choice is likely to increase the consistent use of chosen methods. Counseling also provides information about the benefits of safer sex, increases self-efficacy, and negotiation skills on condom use and promotes disclosure of HIV status [10]. Women who had used contraceptive methods before were 5.6 times more likely to use them currently than those who had no past contraceptive utilization history. Studies done in Ghana and Kenya also found the same result [24, 36]. This might be because previous contraceptive utilization history will help the women in understanding the methods and minimizing the fears concerning the side effects and myths heard, which will make them to choose methods willingly and reduce discontinuation.

A CD4 count of >500cells/mm^3^ showed 2.4 times more tendency of using contraceptive methods than lesser CD4 counts. The finding is similar with that of a research done in Zambia which showed that women who had ≥350cells/mm^3^ had higher odds of contraceptive utilization compared to women who had <250cells/mm^3^ CD4 count. On the other hand the finding was different from that of a study done in Ethiopian region and India. That was because almost 83% and around 65% of the women who had >500CD4 counts either had children or no desire for fertility which might have encouraged them to use the service more than their counter parts [10, 27, 38].

Strengths of the study were: the study covered larger geographic area and used a larger sample size which is representative for the study area and had a good response rate. The study also had some limitations. First there is a risk of social desirability bias, where HIV positive women may over-report their contraceptive use because they are pressured in to practicing safer sex and utilizing the methods by health workers. Second, the study was not triangulated; thus it has not been helped to identify behavioral and psychological factors.

## Conclusion

In this study, utilization of modern contraceptive methods is low relative to similar studies using different studies. Age, residence, counselling by health care providers, discussion with partner, previous contraceptive utilization history and CD4 level > 500cells/mm were found to be associated factors of modern contraceptive utilization. Encouraging discussion with partner and repeated counseling from health care providers may strengthen contraceptive utilization. Moreover attention should be given in the utilization of dual contraceptive methods to simultaneously prevent two undesired outcomes, including unwanted pregnancy and STI transmission. We recommend repeated investigations using larger population based samples that include health care providers and partners whom we have identified as major stakeholders for contraceptive utilization.

## Abbreviations

AOR: Adjusted odds ratio
ART: Anti-retroviral therapy
CD4: Cluster differentiation
EDHS: Ethiopian demographic and health survey
HAART: Highly active anti-retroviral therapy
HIV/AIDS: Human immunodeficiency virus/ acquired immune deficiency syndrome
IUCD: Intra uterine contraceptive device
MTCT: Mother to child transmission
PMTCT: Prevention of mother to child transmission
